# First-Principles Theory of Phase Transitions in IrTe_2_

**DOI:** 10.1021/acs.jpclett.0c00012

**Published:** 2020-02-20

**Authors:** Gabriele Saleh, Sergey Artyukhin

**Affiliations:** Istituto Italiano di Tecnologia, Genoa 16163, Italy

## Abstract

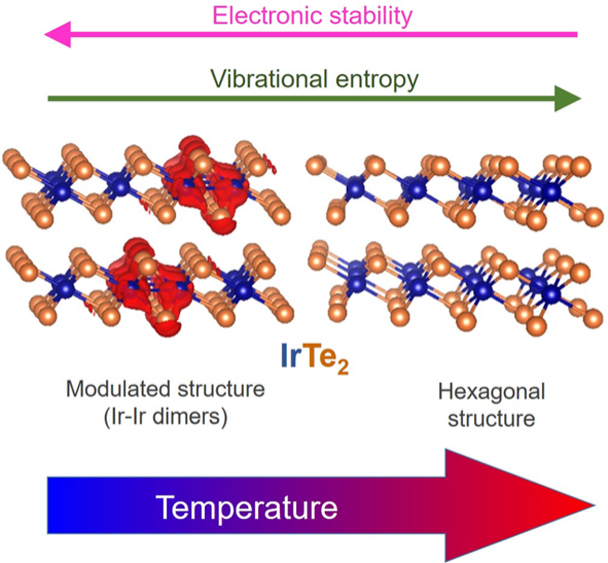

We present a computational study of the electronic structure and lattice dynamics of IrTe_2_ that sheds light on the debated mechanism of the temperature-induced phase transitions of this material. At ambient temperature, IrTe_2_ adopts a hexagonal crystal structure typical of metal chalcogenides. Upon cooling, some Ir–Ir distances shorten, thus inducing lattice modulations. We demonstrate that this is due to the formation of multicenter bonds involving both Ir and Te atoms. We show how the formation of these bonds is energetically favorable but lowers the vibrational entropy; therefore, they are destabilized by temperature. The obtained model is exploited to rationalize the effect of Se doping and other experimental results from the literature.

The coupling among electronic, orbital, and lattice degrees of freedom in materials, and the associated solid–solid phase transitions, give rise to a rich variety of intriguing phenomena such as superconductivity, metal–insulator transitions, and multiferroicity.^1^ Equally rich is the zoo of models developed to rationalize these phenomena,^2^ given their importance for both the fundamental understanding of the matter and the development of new technologies.^3^ Among the materials involved, IrTe_2_ has recently attracted a great deal of interest because of the peculiarity of its temperature-induced phase transitions. Upon cooling, new phases form that are characterized by the shortening (by ∼25%) of certain Ir–Ir bonds, disposed on a regular pattern throughout the lattice,^4^ and by a drop in electrical conductivity.^5^ The phase transitions are of first-order type, with a significant hysteresis.^6^ They were originally thought to originate from charge density waves (CDWs), but it was later recognized that IrTe_2_ lacks the typical CDW signatures such as sinusoidal structure modulation^7^ and band gap opening.^8^ Moreover, the electronic structure rearrangement upon transition extends well outside the Fermi level.^8,9^ Nonetheless, similarly to CDWs, the suppression of bond length alternation (e.g., through doping) leads to the appearance of superconductivity.^10^ The driving force underlying the phase transitions in IrTe_2_ is thus still a subject of debate. Numerous explanations were put forward: in-plane intralayer Te–Te (p orbitals) bond formation,^11^ interlayer Te–Te depolymerization,^7^ Jahn–Teller-like distortion,^12^ Ir charge ordering/disproportionation,^5^ and naturally also Ir–Ir bond formation.^13−15^ Understanding the electronic structure of IrTe_2_, and what factors drive the phase transitions, represents a fundamental step to rationalize these types of phenomena and to exploit them for technological applications.

In this Letter, we present a thorough computational investigation of the electronic structure as well as lattice dynamics of IrTe_2_. We show that while the phases containing short Ir–Ir contacts have a lower internal energy, they are penalized by temperature as their formation lowers the vibrational entropy. The competition between these two factors determines which phase is stable at a given temperature. We study the nature of the Ir–Ir bonds appearing at low temperature through a multitude of tools for chemical bonding analysis, and we unveil their multicenter nature. As to the driving force forming these bonds, we rule out some of the previously put forward hypotheses while reconciling the remaining ones in a unified picture. We also study the phase transition energy paths, whose shape helps explain the observed hysteresis. Finally, we validate the gained insights by applying them to rationalize X-ray absorption and photoemission spectra from the literature and the change in transition temperature upon doping IrTe_2_ with Se (IrTe_2–*x*_Se_*x*_).

At ambient temperature (*T*), IrTe_2_ adopts the 1T structure typical of transition metal dichalcogenides (space group *P*3*m*1; Figure 1a), where Ir is octahedrally coordinated by 6 Te atoms. We shall refer to this phase as “HT”. Note that unlike in the lighter chalcogenide analogues, there is a significant electron sharing among IrTe_2_ layers and the compound is fully metallic.^13^ In the low-*T* phases (*T* < 280 K), the Ir–Ir bond length alternation lowers the symmetry (Figure 1b–d); hence, the reciprocal lattice cell shrinks. Each phase is characterized by the reciprocal space modulation vector **q** = (1/*n*, 0, −1/*n*). We refer to these phases simply as q*n* and to short Ir–Ir bonds as “dimers” (in line with literature nomenclature), even though we will demonstrate that they are actually multicenter bonds. The *n* in the modulation vector above was shown^4^ to vary according to *n* = 3*m* + 2 (*m* = 1, 2, 3, ...). As temperature decreases, *m* increases and so does the density of dimers; that is, first the q5 phase forms, then q8, q11, etc. Instead, when the temperature approaches zero, *n* = 6, and the maximum dimer density is reached. In this study, we consider the HT, q5, q8, and q6 phases,^16^ whose structures were taken from refs (4 and 13).

**Figure 1:**
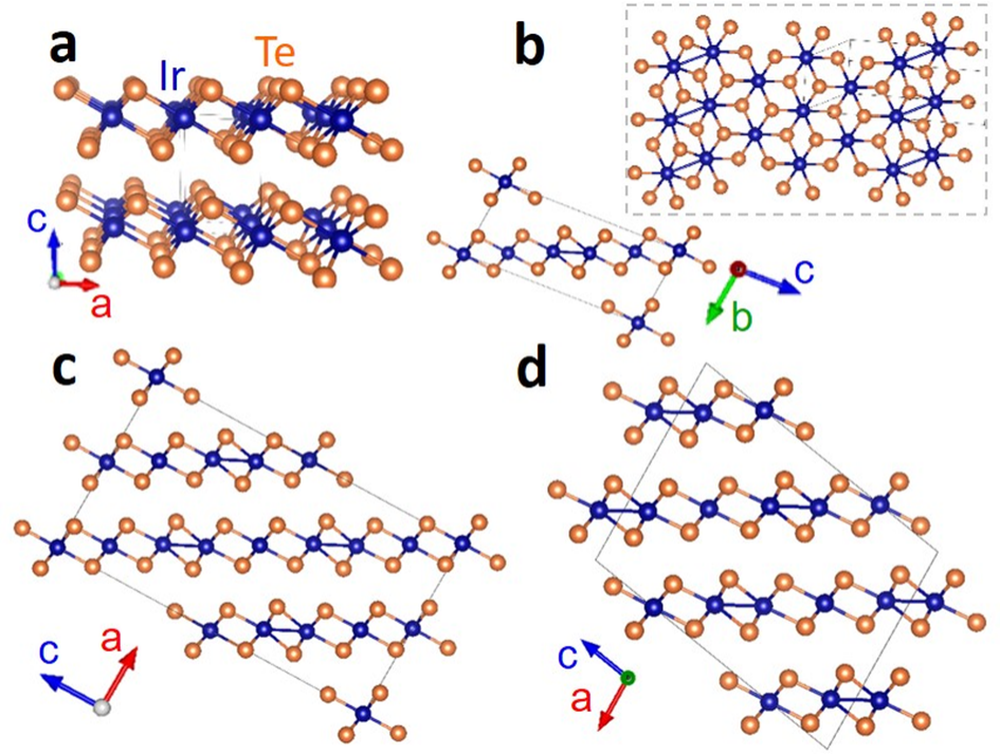
Crystal structures of the phases of IrTe_2_: HT (a), q5 (b), q8 (c), and q6 (d). Unit cells are represented by solid lines. The inset in panel b shows the top-view of one IrTe_2_ layer. In all figures, Ir (Te) atoms are colored in blue (orange), and short Ir–Ir contacts are indicated by blue lines. Structures were drawn with VESTA.^17^

IrTe_2_ is a challenging system for density functional theory (DFT) because the energy differences among phases are in the millielectronvolt per atom range. The situation is further complicated by the presence of strong spin–orbit coupling (SOC). The electronic energy (i.e., the internal energy without the vibrational contributions) is expected to decrease in the order q6 < q8 < q5 < HT; that is, at *T* = 0 K, the phases richer in dimers are more stable (see section S1.2). This trend is notoriously difficult to reproduce with DFT.^4^ In particular, the introduction of SOC is known to destabilize dimers, thereby (over)stabilizing high-*T* phases, leading to a wrong energy ordering when common DFT functionals like LDA, PBE,^18^ and PBEsol^19^ are adopted.^4,13^ This is indeed what we observe (Figure S1). We found instead that the M06L functional,^20,21^ which goes beyond the generalized gradient approximation, produces the correct energy ranking. These results also support the hypothesis that q6 is the ground state of bulk IrTe_2_, which so far has been inferred from surface-sensitive experiments^15^ and/or by analogy with the Se-doped compounds.^4^ When not otherwise stated, the results presented below were obtained with the M06L functional, adopting a plane-wave basis set (VASP code^22^). Section S1 reports a detailed description of the computational strategies and settings.

We evaluated the (harmonic) vibrational partition function of the various phases in order to access their free energies, which govern the thermodynamics of phase transitions (see ref (23) and section S1.4). While the vibrational enthalpy plays a minor role, entropy dictates the stability ranges of the various phases through the TS term (Figures 2a and S3). Our results show that the more dimers a phase contains, the lower its vibrational entropy; that is why temperature destabilizes dimer-containing q*n* phases. Generally, the formation of stronger (stiffer) bonds such as the “dimers” of IrTe_2_ shifts the vibrational modes toward higher frequencies, thereby decreasing the entropy of the system.^24^ This effect is clearly visible in the phonons density of states of IrTe_2_ (Figure 2), and it explains the entropy lowering upon dimer formation. Note that while all our calculations reproduce the formation of dimers as temperature decreases, we found the value of transition temperatures to be heavily dependent on the adopted computational method (Figure S2). This is because small energy changes are involved that are comparable to DFT accuracy, e.g. a 5 meV/atom perturbation shifts the transition temperature by about 250 K (Figure S2). That being said, the qualitative insights discussed above hold true for all the approaches tested (Figure S3). In summary, the stability of the various phases is determined by the competition between the internal energy, favoring the formation of dimers, and the vibrational entropy, destabilizing them. The rise in temperature increases the weight of the latter term over the former.

**Figure 2:**
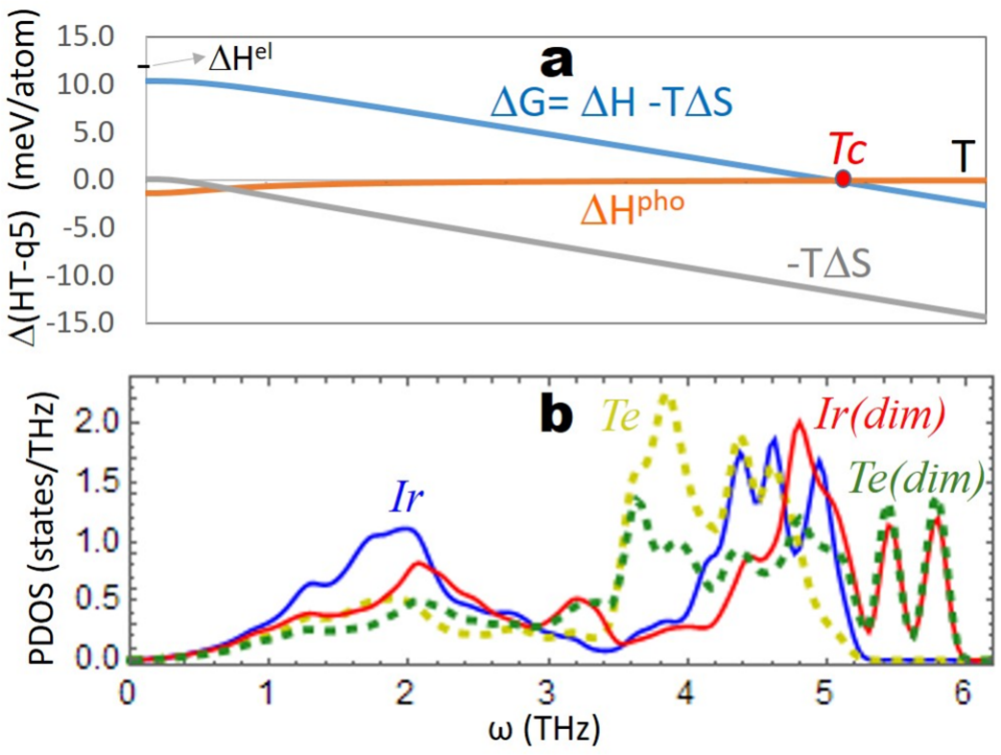
Thermodynamics of the q5-to-HT phase transition of IrTe_2_ at various temperatures. Panel a shows the changes (HT minus q5) in free energy (blue), vibrational entropy (gray), and vibrational enthalpy (orange). Δ*H*^el^ represents the enthalpy difference without vibrational contributions (“electronic energy”). Note that at ambient pressure (*p*), the *p*Δ*V* term is negligible; hence, Δ*H* ≈ Δ*U* (*U*, internal energy; *V*, volume). Tc indicates the temperature of the phase transition. Tc estimations are 280 K (experimental; refs (4) and (5)), 280 K (calculated, PBE, SOC neglected; this work), and 565 K (calculated, M06L; this work). The energies on the left refer to M06L. The plots including the q6 phase are shown in Figure S2. Panel b shows the q5 phonon density of states (PDOS) projected onto Ir and Te atoms. Note how the atoms forming the “dimers” (labeled “dim”) display higher vibrational frequencies. This effect is also visible by comparing the DOS of various phases (Figure S2).

To clarify the chemical bonding pattern of IrTe_2_, we first investigate the role played by interlayer (Te–Te) bonds. We studied the energetics of isolated IrTe_2_ layers (interlayer bonding “switched off”), in HT and q*n* geometries. The formation of dimers in isolated layers is even more favorable than in bulk IrTe_2_ (e.g., Δ*E* for HT-to-q5 transition is −25.9 meV/atom in single layer and −13.5 meV/atom in the bulk; Table S1). Therefore, interlayer Te–Te bonds can be ruled out as the driving force for “dimer” formation. The electronic stabilization of “dimers” is thus to be sought within the layers. To that purpose, we explore the electronic density of states (DOS), its decomposition into atomic orbital contributions (p-DOS), and the partial charge density distributions of isolated layers in the q5 phase (Figures 3 and 4). Five main DOS sections can be identified (labeled with roman numerals in Figure 3), based on the contributing orbitals. Of particular interest is the role of Ir(d) orbitals. The t_2g_/e_g_ splitting can be recognized from the charge density distributions (Figure 4). For t_2g_ states, most of the charge density is localized around Ir, indicating a weak interaction with neighboring atoms. Instead, the (formally) empty e_g_ states display a clear hybridization with Te(p) orbitals. Indeed, regions II and V of the DOS correspond to Ir(d-e_g_)-Te(p) bonding and antibonding states, respectively, as can be inferred from the accumulation (depletion) of charge density in between atoms for states II (V) (see Figures 4c and S10). Note that the interaction of Ir(d-e_g_) with Te(p) orbitals results in bonding states that lie at an energy lower than t_2g_. While the p-DOS of the atoms lying far from the “dimers” is very similar to that of the HT phase (Figure S7), the “dimers” region displays three main differences: lower DOS around the Fermi level (as already noticed in ref (13)), an additional set of empty bands (labeled IV), and more occupied states at the bottom of region II (labeled II′). The charge density of these two additional sets of states is indeed localized in the “dimer” region (Figure 4a,b) and suggests a bonding and antibonding nature for states II′ and IV, respectively. Moreover, the two bridging Te atoms are clearly involved in the chemical bonding. The “dimer” can thus be identified as a 4-center Ir_2_Te_2_ bond formed by Ir(d) and Te(p) orbitals. This multicenter nature is confirmed by the lack of a direct Ir–Ir bond when the charge density topology is analyzed in the framework of the quantum theory of atoms in molecules^25^ (Figure S12). Note that while we studied isolated IrTe_2_ layers for their neater bonding features, bulk DOS differs only by the peak broadening due to interlayer interactions (Figure S6). It is noteworthy that the DOS from PBE calculations neglecting SOC displays the same qualitative features as in Figures 3 and 4 (Figures S8 and S9).

**Figure 3:**
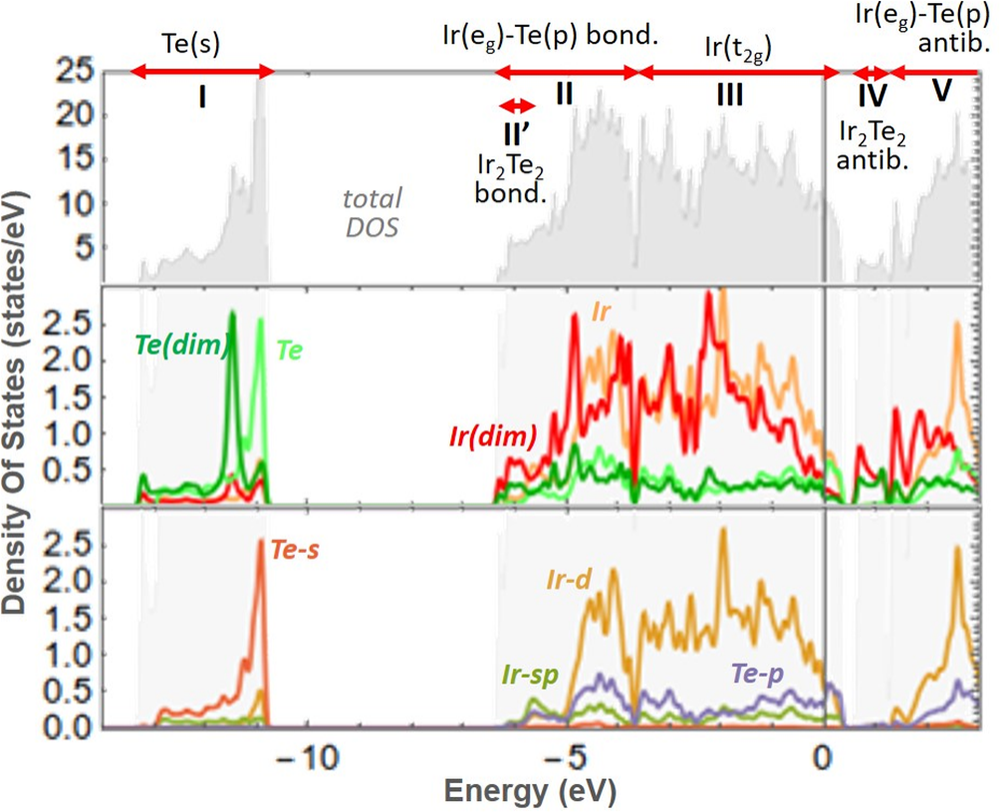
Density of states for an isolated layer of IrTe_2_ in q5 phase. The total DOS is shown in gray in the top panel. The red arrows represent the DOS sections discussed in the main text, identified by roman numerals. by roman numerals. The middle and lowest panels report atom and orbital projections (p-DOS), respectively. “(dim)” indicates the p-DOS of atoms forming the “dimer”. Note how sections II′ and IV are missing in the lowest panel, which displays the orbital projections of atoms far from the “dimers”.

**Figure 4:**
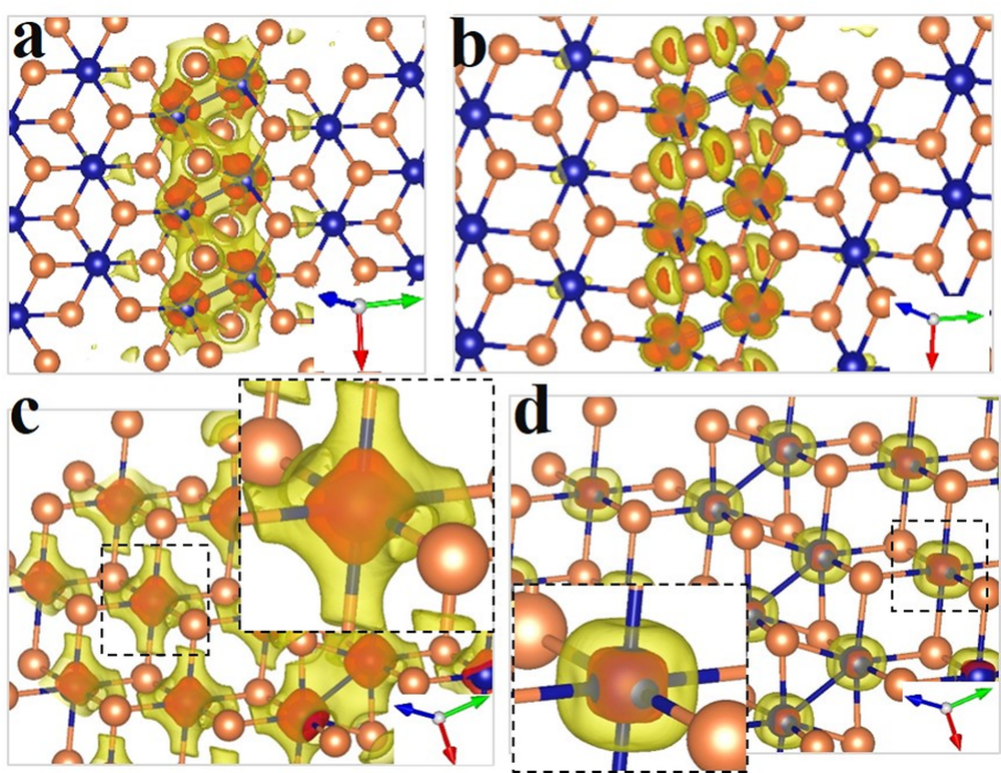
Charge density distribution of selected states of IrTe_2_ in q5 phase. Each panel shows the distribution relative to the states of a given energy window labeled in Figure 3, namely, (a) −6.75/–5.75 eV (section II′ in Figure 3), (b) +0.4/+1.3 eV (section IV), (c) −6.5/–3.5 eV (section II; enlargement of the dotted box in the inset), and (d) −3.5/0.4 eV (section III; enlargement of the dotted box in the inset). In each panel, two isosurfaces are shown: red (higher value) and yellow (lower value). Isovalues and additional plots, including two-dimensional maps can be found in Figures S10 and S11. Note in panel c (d) the octahedral (cubical) shape of the charge density around Ir, typical of the charge density distribution of e_g_ (t_2g_) d orbitals.^26^

The above chemical bonding scenario can be put on energetic grounds by the analysis of the crystal orbital Hamilton population (COHP).^27,28^ COHP partitions the energy of electronic states into bonding and antibonding contributions from each atom pair (negative and positive COHP, respectively), giving rise to DOS-like plots (Figure 5c). The COHP results fully confirm the previous bonding–antibonding assignments and the involvement of Te orbitals in the “dimer”, manifested as a significant Ir–Te COHP value in the “dimer” regions of the DOS. ICOHP, i.e., the integral of COHP for all occupied states, measures the contribution of a given bond to the electronic energy of the crystal.^29^ In absolute ICOHP value, Ir–Te is the dominating interaction in IrTe_2_ (Figure 5). However, what matters for the phase transition energetics is the change in ICOHP, which is shown Figure 5a. As expected, the main stabilization of the q5 structure comes from the Ir–Ir and Ir–Te bonds forming the “dimer” and from the Te–Te interaction just above it. This further confirms that “dimers” are not simply Ir–Ir bonds. Interestingly, the most destabilized bonds are the Ir–Te next to the “dimers” (Figure 5a). This explains why even in the (low-*T*) ground state of IrTe_2_ there is an alternation of long and short Ir–Ir bonds, which is reminiscent of Peierls distortions. In order to achieve the overall electronic energy lowering, the formation of “dimers” requires the lengthening and consequent destabilization of the neighboring bonds. A hypothetical shortening of adjacent Ir–Ir bonds would thus be energetically unfavorable.

**Figure 5:**
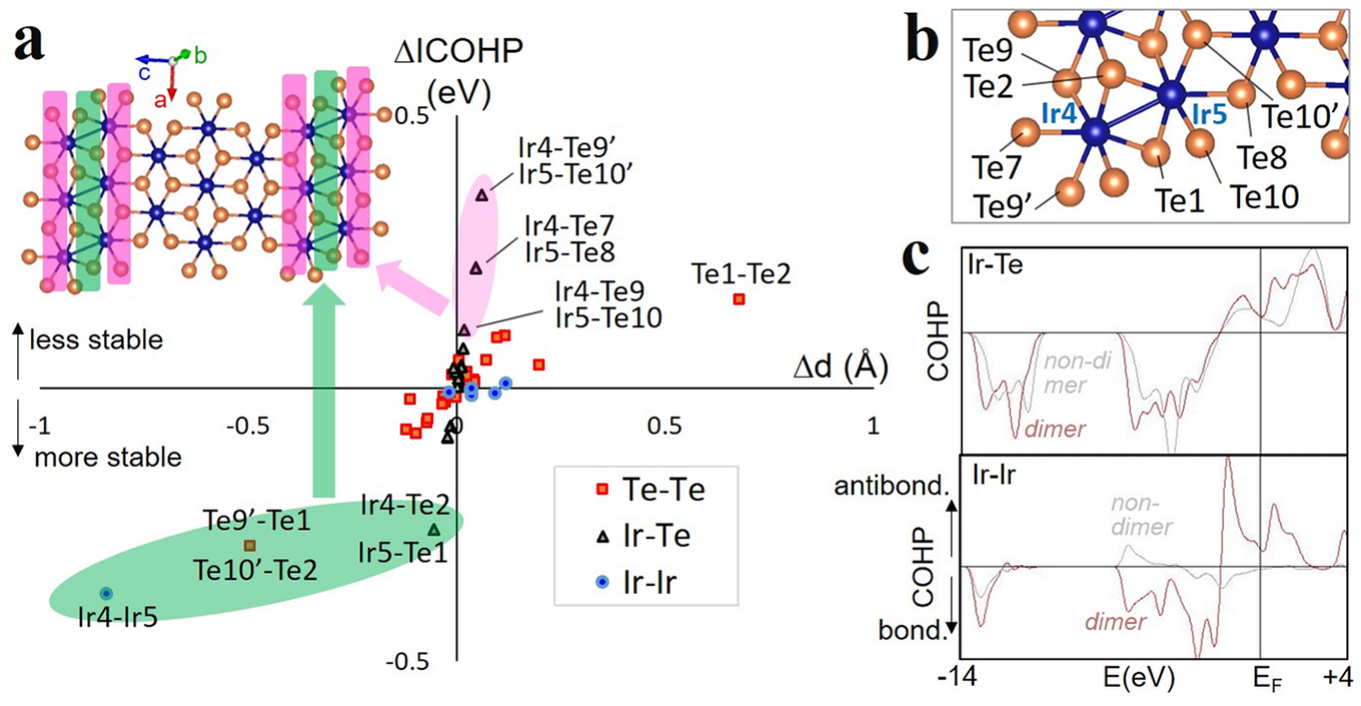
Crystal orbital Hamilton population of bulk IrTe_2_. (a) Plot whose axes represent, for each bond in the unit cell, the change in ICOHP^30^ (ΔICOHP) and bond lengths (Δ*d*) when passing from HT to q5 phase. Each bond type is labeled with a different color, which is shown in the bottom right legend. The average ICOHP values for each bond type in q5 phase are −0.19 eV (Ir–Ir), −2.5 eV (Ir–Te), and −0.15 eV (Te–Te). Bonds relevant for the discussion are labeled in the plot, and their position is shown in panel b. The top-left inset shows in a pictorial way that in the q5 phase, the bonds within (next to) the “dimers” are stabilized (destabilized) with respect to the HT phase: those bonds that in the plot are enclosed in the green/violet shaded area are located in the crystal in the region shaded by the same color. Panel c reports two representative COHP plots: Ir–Ir and Ir–Te. The bonds within the “dimer” are distinguished from those that are distant from it (in the case of Ir–Te, Te is the atom bridging the “dimer”, i.e., Te1 and Te2 in panel b). Additional COHP plots are presented in Figure S4.

From the kinetic point of view, nudged elastic band (NEB) calculations indicate the presence of a barrier along the phase transitions (Figure S5); that is, a higher-energy transition state is to be formed in order for “dimers” to build or break. For first-order transitions, the NEB reaction path can be viewed as simulating the growth of the forming phase domain. The height of the barrier (66 meV/cell for HT-to-q5) is comparable to and greater than the *k*_B_*T* thermal energy at the transition temperature. This qualitatively explains the observed hysteresis, although additional factors may hide in the complex nucleation process. Along the NEB phase transition (minimum-energy) path, all Ir atoms move in a concerted way (Figure S5e), thus supporting the mechanism sketched by Mauerer et al.^31^ They showed that the perturbation induced by one Ir–Ir “dimer” breaking extends for several neighboring Ir–Ir pairs, a fact that was used to explain the domain propagation anisotropy observed through atomic-resolution microscopy experiments. We note that the height of our NEB barrier is close to the one obtained in ref (32) with a different approach.

The insights gained above for IrTe_2_ can be applied to rationalize the behavior of the Se-doped compound, IrTe_2–*x*_Se_*x*_ (*x* = 0.10 for q5 and *x* = 0.04 for q6). Se-doping favors dimer formation, thereby raising the transition temperatures.^15^ Our calculations capture this doping effect. They indicate a preference for Se to lie in the “dimer” position (Te1/Te2 of Figure 5b), although in q6 the possible substitution positions are close in energy (Δ*E* = 1.5 meV/atom, Table S2). In fact, X-ray diffraction experiments of Pascut et al.,^4^ carried out on a sample with higher Se dopant concentration (IrTe_1.6_Se_0.4_), did not show any preferential Se distribution in the q6 phase. Our calculations reveal that Se stabilizes q*n* phases through the electronic energy term [Δ*H*_el_ of Figure 2]. This effect can be explained by considering the orbital sizes:^32,33^ 4p (Se) orbitals are smaller than 5p (Te) orbitals; hence, they overlap better with the even smaller 5d (Ir) orbitals. A greater overlap guarantees a larger stabilization of the Ir–Se bonding states compared to Ir–Te. This is confirmed by Ir–Se ICOHP values being more negative than Ir–Te and by the further shortening of the Ir–Ir “dimer” distance upon Se doping (Table S3). Finally, our NEB calculations show a substantially barrierless phase transitions for IrTe_1.90_Se_0.10_, consistent with the observed hysteresis quenching upon Se doping.^4^

Finally, we discuss the hypotheses and experimental results from the literature on IrTe_2_ in light of the insights gained in the present study. Our chemical bonding analysis rules out Te–Te bonds and Jahn–Teller distortion as driving forces for “dimer” formation. Other authors proposed a Ir^3+^/Ir^4+^ charge ordering, the “dimer” Ir being more positively charged. This model follows from the calculated Ir atomic charges in q*n* phases^12,13^ and from the observed photoemission spectra, showing a split of the Ir-4f signal upon dimer formation.^5^ Both these observations, however, can be simply explained by the electronic rearrangement taking place on those Ir atoms that form “dimers” (Figures 3 and 4). In particular, atomic charges are evaluated by integrating the charge density inside spheres of defined radius, generally nonoverlapping. It is thus obvious that this population diminishes if more charge is accumulated between atoms (Figure 4). The present study adds on to the idea of Ir–Ir bond formation, demonstrating their multicenter nature and showing why they break at high temperature. The Te(p)-Ir(d-e_g_) bonding we unveiled is reminiscent of the “ligand hole” concept used to rationalize the structure of AuTe_2_^34^ and AgAgTe_4_.^35^ The involvement of Te(p) orbitals in the “dimers” was also hypothesized by Takubo et al.^9^ Interestingly, their X-ray absorption experiments (Figure 2c of ref (9)) show that the “dimer” formation depletes the DOS just above the Fermi level and creates additional empty states at slightly higher energies, +1.3 eV. These results fully agree with our calculated DOS, which also allow us to identify those additional states at 1.3 eV as the antibonding orbitals relative to the “dimers” (Figures 3 and 4).

In conclusion, we presented a model for the debated mechanism of IrTe_2_ phase transitions. We have shown that the temperature ranges of stability for the various phases are determined by the competition between electronic (internal) energy and vibrational entropy. We demonstrated that the so-called “Ir–Ir dimers” forming at low temperature are in fact multicenter bonds. Se-doping was simulated and its effect explained. Our model can also rationalize X-ray absorption and photoemission spectra from the literature. Besides elucidating the hitherto poorly understood mechanism of IrTe_2_ phase transitions, the present study may be considered as a fresh point of view that can be helpful to rationalize those solid–solid phase transitions that cannot be satisfactorily explained by commonly adopted models, such as CDWs or Mott transitions.

## Supporting Information Available

Detailed description of computational methods, results obtained with other DFT functionals, NEB results, and details of the electronic structure of pure and Se-doped IrTe_2_ (PDF)
