# Supplementary material for: First-Principles Theory of Phase Transitions in IrTe2
Source: J Phys Chem Lett. 2020 Feb 20;11(6):2127–32. doi: 10.1021/acs.jpclett.0c00012 (PMC7997575; doi:10.1021/acs.jpclett.0c00012)
Supplement: Supplementary file 1 — jz0c00012_si_001.pdf [file jz0c00012_si_001.pdf]

First-Principles Theory of Phase Transitions in $IrTe_2$

Gabriele Saleh[1,*], Sergey Artyukhin[1]

[1] Istituto Italiano di Tecnologia, Genoa 16163, Italy

*Email: gabrielesaleh@outlook.com

Supporting Information

Table of Contents

S1 Computational Methods: detailed description 3

S1.1 General computational settings 3

S1.2 Choice of DFT functional and stability of $IrTe_2$ phases. 4

S1.3 Simulations of Se doping 5

S1.4 Phonon calculations 5

S1.5 Nudged Elastic Bands (NEB) calculations 6

S1.6 Crystal Orbital Hamilton Population (COHP) 6

S2 Additional results of reaction energies 8

S3 Additional density of states and charge density maps 12

S4 Results of Se-doping 16

S1 Computational Methods: detailed description

S1.1 General computational settings

All the calculations were carried out with Density Functional Theory (DFT) Hamiltonians through the VASP code[1] and in the framework of the all-electron projector augmented wave (PAW) method[2]. When not otherwise specified, the following settings were adopted. The plane wave kinetic energy cutoff was set to 500 eV and the reciprocal space was sampled through a uniform, Γ-centered grid with 0.20/2π $Å^{-1}$ point spacing. The Self Consistent Field procedure was considered converged when the energy difference between two consecutive cycles was lower than $5*10^{-6}$ eV, while structure optimization runs were stopped when the energy difference between one step and the previous one was lower than $5*10^{-5}$ eV. In all structural optimizations, both the atomic positions and the unit cell shape and volume were optimized, *i.e.* they were changed until the minimum energy was reached. To minimize Pulay stress, geometry optimizations were restarted (from the minimum-energy geometry) until no cell edge changed by more than 0.5 %. The PAW pseudopotentials adopted were those recommended by VASP[3], including that of Selenium for the doped compound. The choice of DFT functional is discussed in the next section. As commonly done in solid state, we adopted smearing functions on the bands occupation in order to make the SCF procedure more stable and faster to converge. We adopted the Methfessel-Paxton order 1 method ("ISMEAR=1" tag in VASP) for all the calculations except DOS, checking that the (artificial, electronic) 'entropy' of the system was always negligible (< 1 meV/atom). For DOS calculations, we used the tetrahedron method with Blöchl corrections ("ISMEAR=-5" tag in VASP), as suggested by VASP manual. The resulting DOS, however, displayed narrow peaks for isolated layer. Therefore, for convenience of representation, we applied a smearing function to the DOS of Fig. 3 of the main text. In particular, we convoluted the DOS with normalized Gaussians ("GaussianFilter" option of Matlab). The original, non-smeared DOS is shown in Fig. S10. Concerning the number of points used for the fast Fourier transform, we adopted the 'prec=accurate' VASP settings. However, for M06L, it was necessary to double the number of FFT points with respect to the 'prec=accurate' settings in order to achieve SCF convergence.

Since for plane wave calculations the cell dimension determines the size of the basis set, and considering that the cell of HT is much smaller than those of qn phases (HT, q5, q6 and q8 contain 3, 15, 36 and 48 atoms, respectively), we performed the calculations on the HT structure a q5-like, 15-atom supercell. This q5-like cell was obtained by applying the transformation matrix $\begin{bmatrix} 1 & -1 & 2 \\ 0 & -1 & 4 \\ 0 & 1 & 1 \end{bmatrix}$ to the original HT cell. The energy difference (PBE functional, spin-orbit coupling neglected) between HT and q5 was found to change by a small yet non negligible amount: from 16.1 meV/formula-unit (HT in its primitive cell) to 18.1 meV/formula-unit (HT in q5-like cell), *i.e.* an 11% change. The same procedure can be applied to the energy difference between HT and q6: the HT cell can be made q6-like by applying the transformation matrix $\begin{bmatrix} 1 & 1 & 2 \\ 2 & 0 & 4 \\ -2 & 0 & 2 \end{bmatrix}$. The HT-q6 energy difference changes by a comparatively small amount when passing from q5 to q6 cell: the energy difference changes from 26.6 meV/formula-unit (HT in q5 cell) to 25.2 meV/formula-unit (HT in q6 cell), *i.e.* a 5% change. In light of this, we adopted the q5-like cell for HT to calculate all energy differences as we found it good enough to avoid significant changes in the energy differences due to the change in the basis set.

Note that the 500 eV plane-wave cutoff is considerably higher than the highest default value associated to the VASP pseudopotentials adopted (211 eV). Indeed, the energy differences discussed above are very small in absolute value (< 1 mev/atom): they are only significant in percentage as the energy differences among $IrTe_2$ phases are small. Finally, we note that by calculating the energy of qn phases with respect to HT with a considerable smaller plane-wave cutoff of 211 eV, we observe the following changes compared to the 500 eV results (M06L functional, spin-orbit coupling included): q5 changes from -13.5 meV/atom (500 eV cutoff) to -11.8 (211 eV cutoff) ; q8 from -17.5 meV/atom to -15.4 meV/atom ; q6 from -24.0 meV/atom to -21.2 meV/atom. In light of these results we can conclude that i) with 500 eV plane-wave cutoff, the energy differences among phases are already well-converged ii) adopting a 211 eV cutoff, as we did for the most expensive calculations such as phonons with M06L+SOC (see infra), provides a good approximation to the 500 eV results. This is especially true for those calculation where the unit cell does not change (phonons within the harmonic approximation) or changes only slightly (nudged elastic bands).

Calculations on isolated layers were performed by first applying a $\begin{bmatrix}1 & 0 & 0\\ 0 & 1 & 1\\ 0 & -1 & 1\end{bmatrix}$ trasformation matrix (assuming that the direction perpendicular to the layers is the *bc* diagonal), so that the $IrTe_2$ layers are parallel to one cell edge. Then all the layers but one are removed from the unit cell. This procedure gives a layer-layer distance of at least 10 Å, hence interlayer interaction can be considered negligible, especially concerning orbitals hybridization (or "interlayer hopping", to put it in physicists' terms).

S1.2 Choice of DFT functional and stability of $IrTe_2$ phases.

A necessary condition for the $IrTe_2$ phase transitions to be reproduced is that the electronic energy (defined as the internal energy without the vibrational contributions) of the various phases follow the order HT>q5>q8>q6 . This is because the vibrational contribution to the free energy follows the opposite trend (q6>q8>q5>HT), *i.e.* it destabilizes the phases that are more rich in dimers. The higher the temperature, the stronger this effect (see main text and Fig. S2). It is the subtle balance between electronic and vibrational contributions that determines the stability ranges of the various phases (Fig. S2). Therefore, if one of the phases does not follow the abovementioned electronic energy ranking, it is automatically predicted to be unstable at all temperatures, independently from the magnitude of its vibrational contribution.

It is known in literature (*e.g.* ref 4) that the expected electronic energy trend cannot be reproduced with LDA or GGA functionals when spin-orbit coupling (SOC) is included. Our calculations with PBE[5] and PBEsol[6] confirmed this deficiency. We therefore decided to move one step up in the Jacob's ladder of DFT functionals[7], by adopting a meta-GGA functional. We chose M06L[8], which was shown to outperform in accuracy all other functionals by a large margin when it comes to compounds containing transition metals[8]. Indeed, the M06L functional was able to reproduce the expected energy ordering of $IrTe_2$ also when SOC was included. The results are shown in Fig. S1. Note that when SOC is neglected, all the functionals produced the expected energy order, *i.e.* q6<q8<q5<HT.

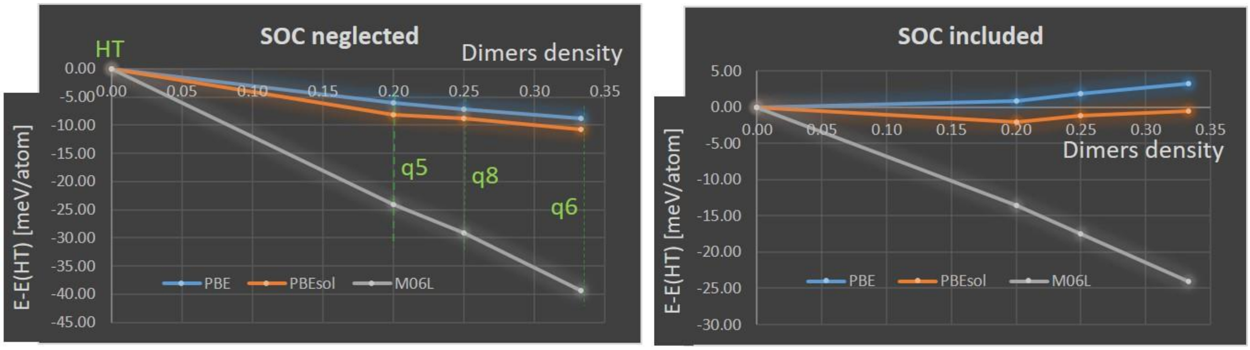

Figure S1 Energetics of $IrTe_2$ phases with different DFT functionals with (right) and without (left) Spin Orbit Coupling. Energies are calculated with respect to the HT phase. 4 phases were considered: HT, q5, q6, and q8 (see main text for structural description). To each of them we assign a density of 'dimers' calculated as: "Dimers density"= number of short Ir-Ir bonds/number of Ir atoms in the unit cell.

S1.3 Simulations of Se doping

Se doping is known to be substitutional[9]. To achieve the desired doping concentration of 5% or less, we doubled the unit cell in the shortest direction, so as to avoid the interaction of atoms with their periodic image. One Te atom of the cell is then substituted by Se. This leads to $IrTe_{1.90}Se_{0.10}$ for the q5 phase and $IrTe_{1.96}Se_{0.04}$ for q6. As to the possible substitution position, in q5 we consider 3 positions: 'dimer' (the Te atom that bridges the two Ir forming the dimer, i.e. Te1/Te2 in Fig. 5 of the main text), 'next to dimer' (Te7/Te8 of Fig. 5 in the main text), and 'far from dimer' (the Te atom(s) farthest from the short Ir-Ir bond). In q6, the 'next to dimer' position is also the farthest from the dimer, hence only two substitution positions were simulated.

S1.4 Phonon calculations

All the phonon calculations were carried out through the PHONOPY code[10] (finite displacement method) adopting the harmonic approximation. Basic thermodynamics establishes that the macroscopic vibrational thermodynamic quantities can be calculated from the vibrational partition function (the following expression makes use of the Bose-Einstein distribution to express the mean occupation number of a vibrational given state):

$$Z = \prod_{qv} \frac{e^{-\frac{\hbar\omega(\mathbf{q}v)}{2kT}}}{1 - e^{-\frac{\hbar\omega(\mathbf{q}v)}{kT}}} \quad ,$$

where **q***v* indicates the *v*-th branch of the phonon with wavevector **q**, ω is the frequency, k the Boltzmann constant, and T the temperature. In particular, the vibrational Helmotz free energy is calculated as:

$$F = -kT\ln Z \ .$$

The total free energy is then obtained by adding the electronic energy (*i.e*. the energy resulting from the static DFT calculation) to the vibrational contribution. Note that in the text we label the free energy as G (Gibbs free energy), as we use it to approximate the free energy at constant pressure, which corresponds to the experimental conditions most commonly adopted. However, as we adopt the harmonic approximation, the unit cell is kept frozen when calculating the phonons (constant volume). Therefore, technically speaking this would be the Helmotz free energy.

From a computational point of view, we calculated the force constants needed for phonons evaluation on a 3x2x1 supercell for q5 and HT phases (HT phase being transformed in a q5-like cell, see Section S1.1), and a 3x1x1 cell for q6. We adopted a 32x32x32 q-point mesh, which is already well converged, i.e. it shows negligible changes in the free energy with respect to the 16x16x16 mesh. Given the high computational cost of M06L+SOC calculations, the corresponding vibrational properties were calculated with a plane-wave energy cutoff of 211 eV. This value is the maximum energy cutoff suggested for the adopted PAW pseudopotentials. Considering the discussion in Section S1.1, and the fact that all the forces are calculated on the same unit cell and similar geometries (error cancellation can be anticipated), we expect the cutoff lowering to produce negligible differences in the vibrational properties with respect to the 500 eV cutoff. We observed no sizeable imaginary frequencies (except for transition states, where they are expected, see below), confirming that all the considered structures are true minima on the potential energy surface of $IrTe_2$.

S1.5 Nudged Elastic Bands (NEB) calculations

Nudged Elastic Bands (NEB) calculations. Climbing-Image, Variable-Cell NEB (CI-VC-NEB) calculations were performed by generating 5 intermediate structures between the reactant and the product (*i.e.* two IrTe2 phases), for a total of 7 images. The initial images were generated by the "nebmake" script[11]. The 'climbing image' method[12] is chosen to ensure that one of the images is the saddle point connecting reactant and product, thereby having an accurate estimation of the reaction barrier. The spring constant among images was set to 5.0 eV/Å^2, and the NEB convergence threshold was set to $1*10^{-5}$ eV. Note that NEB calculations could be performed only for the transitions between HT and low-temperature phases. This is because the transitions between low-temperature phases would require the use of computationally unaffordable unit cells. For example, the q5-to-q6 transition is to be simulated in a unit cell that can describe both q5 and q6 modulations; such a cell would contain 540 atoms. Nonetheless, the fact that HT-to-q5 and HT-to-q6 display kinetic barriers of similar magnitude (Fig. S5; the one for q6 is greater due to the high number of 'dimers' in the cell) suggests that a similar barrier would be found for the q5-to-qn (n > 5) transition. Note that the bare CI-VC-NEB results include only the electronic energy (*i.e.* the internal energy without vibrational contribution), thereby neglecting temperature effects. To have a more realistic estimation of the barrier at the transition temperature, free energies have to be calculated, as explained below.

By calculating the NEB transition path, we simulate in an approximate fashion the growth of the domain of the forming phase (as mentioned in the main text). In other words, the NEB barrier represents the kinetic barrier for the reaction event of one unit cell of the reactant phase transforming in the product phase. The probability of these types of processes to take place can be described within the models of Eyring[13] or Vineyard[14]. According to Eyring, the reaction rate k_r is expressed as:

$$k_r = \frac{k_B T}{h} e^{-\Delta G^{\neq}/k_B T} = \frac{k_B T}{h} e^{\Delta S^{\neq}/k_B} e^{-\Delta H^{\neq}/k_B T}$$

Where $\Delta A^{\neq}$ is the difference in the thermodynamic quantity A between the transition state and the reactant. G, H, and S represent the free energy, the enthalpy, and the entropy, respectively. T is the temperature, k_B and h are the Boltzmann's and Planck's constants, respectively. The vibrational contribution to $\Delta G^{\neq}$ is calculated by excluding from the transition state partition function the phonon branch having imaginary frequencies. The results are shown in Fig. S5.

S1.6 Crystal Orbital Hamilton Population (COHP)

COHP contributions were evaluated with the code Lobster[15]. In order to obtain the differences in ICOHP for each bond, we performed calculations on HT (in the q5 unit cell) and q5 phases adopting the same

atom numbering in both cases. Then the ICOHP differences were calculated with an in-house built fortran code. The bonds included in the plot were those whose length fall within the following cutoffs: 4.5 Å, 3.5 Å, 4.8 Å for Ir-Ir, Ir-Te, and Te-Te, respectively. As the lobster code cannot read wavefunctions obtained from non-collinear spin calculations (that are necessary to include SOC), COHP was calculated using the PBE functional without SOC. This choice is justified energetically by the error compensation between using PBE (instead of M06L) and neglecting SOC (Fig. S1). As to the electronic structure, the DOS and charge distribution of PBE without SOC are qualitatively equivalent to those of M06L with SOC (Figs. S8-S11). Therefore, the qualitative conclusions drawn from COHP analysis at the PBE-noSOC level of theory will apply to the M06L with SOC as well. Note that the sum of ICOHP contributions for all atom pairs, including on-site terms, gives the 'band energy', that is the sum of the energies of all occupied states. In other words, the integral of E*DOS(E) up to the Fermi level. The band energy is generally not the same as the total electronic energy, but it is closely related to it[16,17].

S2 Additional results of reaction energies

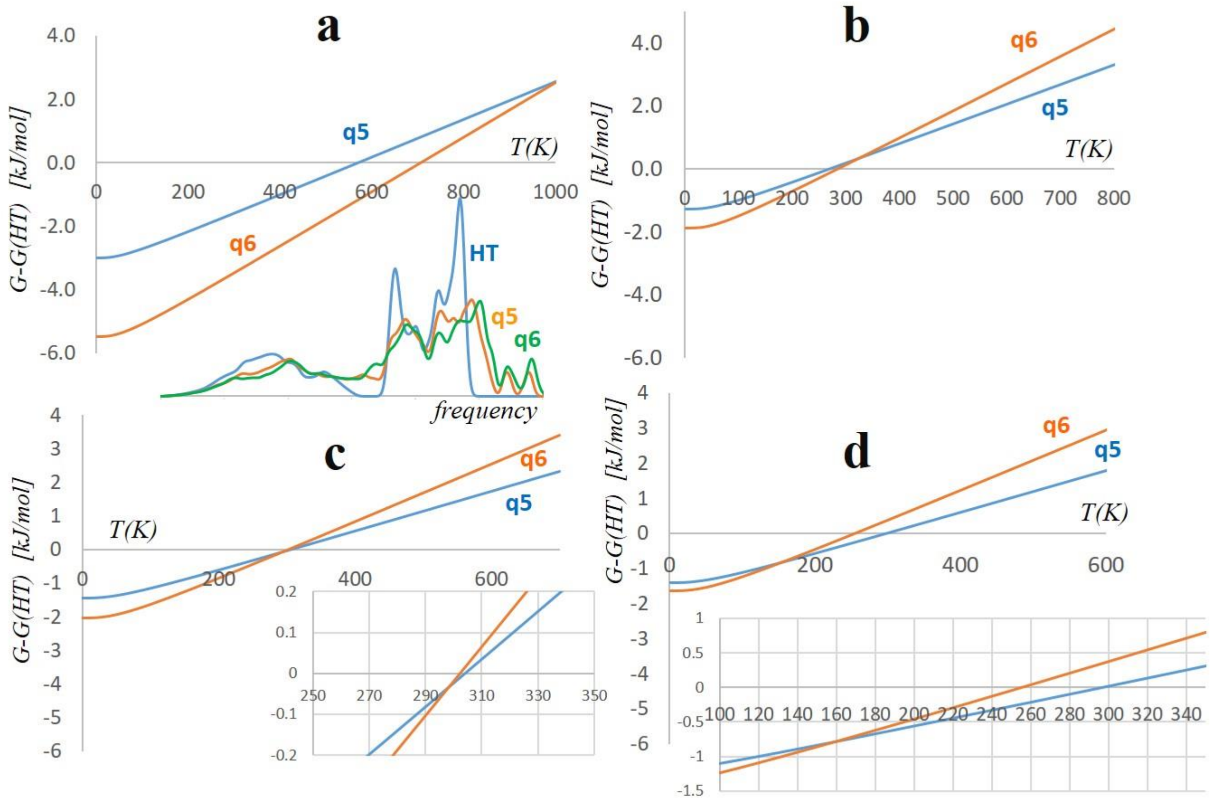

Figure S2 Free energies (G) of $IrTe_2$ phases as a function of temperature calculated with different methods. All the plots report the energy of q5 and q6 phases with respect to the HT phase. Methods adopted: a) M06L, Spin-Orbit Coupling (SOC) included. b) PBE, SOC neglected. c) electronic energy calculated with PBE (SOC neglected), vibrational contributions calculated with M06L (SOC included). d) same as panel a, but with an arbitrary change in the electronic energy of +5.5 meV/atom (1.6 kJ/mol) for the q5 phase and +12.8 meV/atom (3.8 kJ/mol) for q6. Note how relatively small energy perturbations can change the transition temperatures by hundreds of Kelvin: from completely off (a) to very close (d) to the experimental estimates. In panels c and d, the inset shows an enlargement near the transition temperatures, while in a) it shows the phonons density of states for the various phases, normalized for the number of atoms. Note that the kJ/mol energies refer to 1 mol of $IrTe_2$.

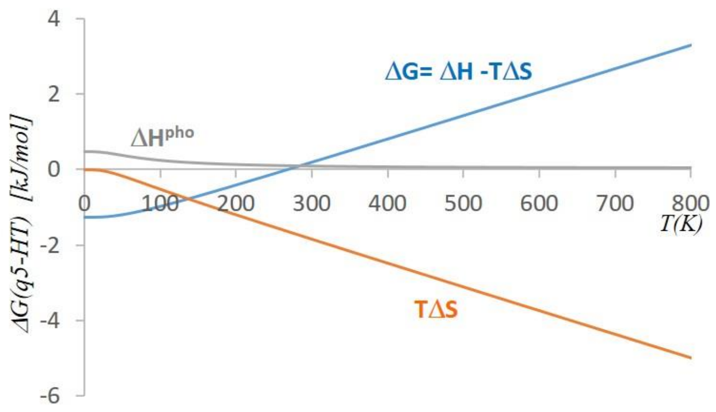

Figure S3 Free energy of HT→q5 reaction calculated with PBE functional (SOC neglected) decomposed into enthalpic and entropic contributions, similarly to Fig. 2 of the main text, except that in the main text the back reaction q5 →HT was considered.

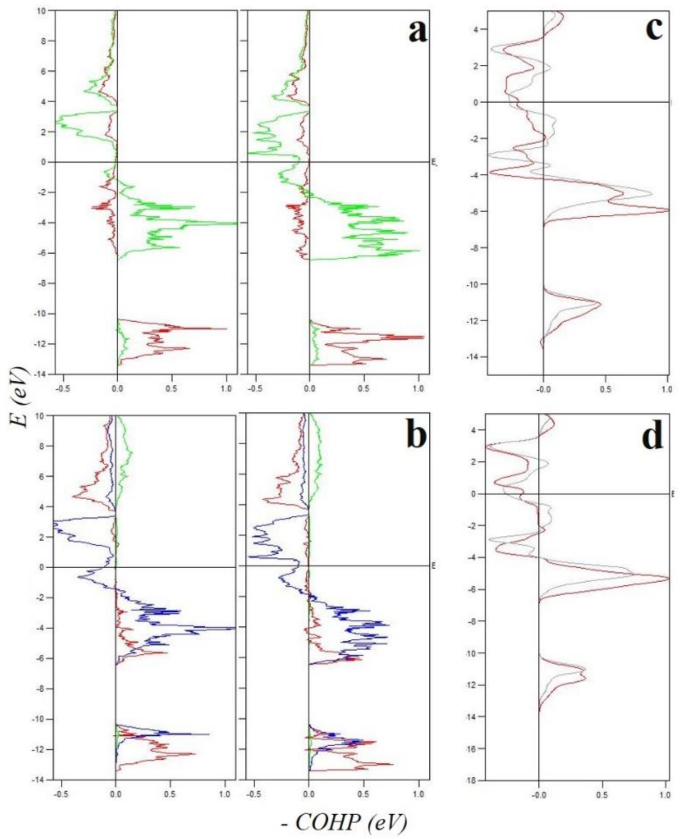

Figure S4 Crystal Overlap Hamilton Population for various bonds in the q5 phase of $IrTe_2$ (PBE functional, SOC neglected). a,b) Ir-Te interactions decomposed into orbital contributions. The right (left) plot shows the bonds inside (far from) the 'dimer' (e.g. Ir4-Te1 of Fig. 5 of the main text is a Ir-Te bond inside the dimer). In (a), we show the interaction of all the orbitals of Ir with the Te-s orbitals in red and with the Te-p orbitals in green. In (b), we show the interaction of all Te orbirals with Ir-s orbitals in green, with Te-p orbitals in red and with Ir-d orbitals in blue. Panels c and d report the COHP of Te-Te interactions. In particular (c) is for intralayer bonds (specifically bonds parallel to the layer plane) and (d) for interlayer. The red curve refers to the atoms closest to the shortest Ir-Ir contact, while grey lines indicate atoms farthest from the Ir-Ir contact. Note that on the horizontal axis, it is the quantity -COHP that is plotted, hence positive values (right part of each graph) indicate bonding contributions.

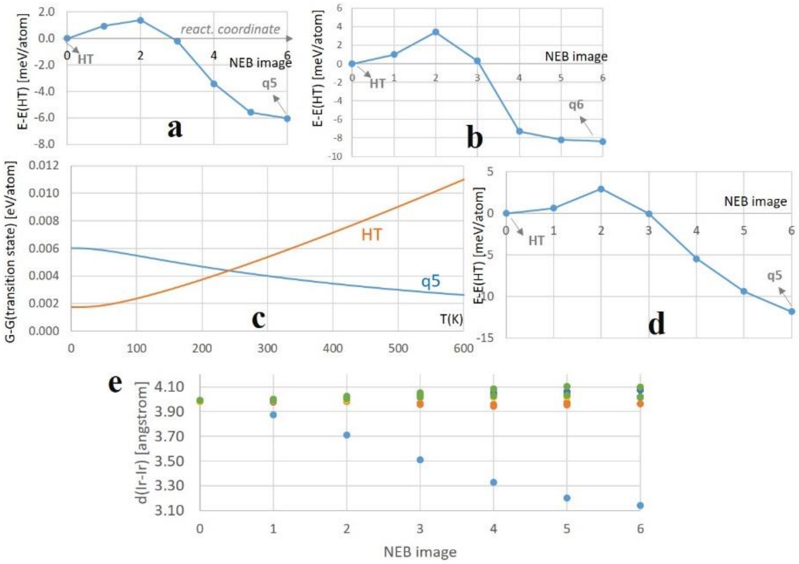

Figure S5 Nudged elastic Bands results for HT-to-q5 (a) and HT-to-q6 (b) transitions, calculated with PBE functional neglecting SOC (PBE-noSOC). The energetics of the transition path is obtained through a 7 images CI-NEB calculation. Note that for HT-to-q6 transition, due to the high computational demand, we set a less tight convergence threshold of $1*10^{-4}$ eV, the same being true for the M06L+SOC calculation (see below). As NEB calculations are interpreted only in a qualitative way, the lowering of the convergence threshold is expected to have negligible effects. Panel c reports the free energy barrier (calculated as described in Section S1.5) for the q5-to-HT transition as a function of temperature, indicated as free energy difference between the transition state (NEB image n 2 in panel a and HT phase (orange curve) and between the transition state and q5 (blue curve). Panel d shows the HT-to-q5 NEB path calculated with M06L including SOC (lower cutoff, as for phonons, see Section S1). These M06L+SOC calculations are included to show that the qualitative picture remains the same when more accurate functionals are adopted. Panel e shows the change of Ir-Ir distances along the NEB path shown in panel a. Each point represents one Ir-Ir distance (cutoff 4.1 Å) in the unit cell.

Table S1 Energies of phase transitions from the HT phase to q5 and q6 phases for isolated layers of $IrTe_2$. The atomic positions in the single layers were kept frozen at the positions obtained in bulk optimization.

phase transition	ΔE M06L plus SOC (meV/atom)	ΔE PBE no SOC (meV/atom)
HT → q5	-25.9	-24.3
HT → q6	-49.9	-51.5

S3 Additional density of states and charge density maps

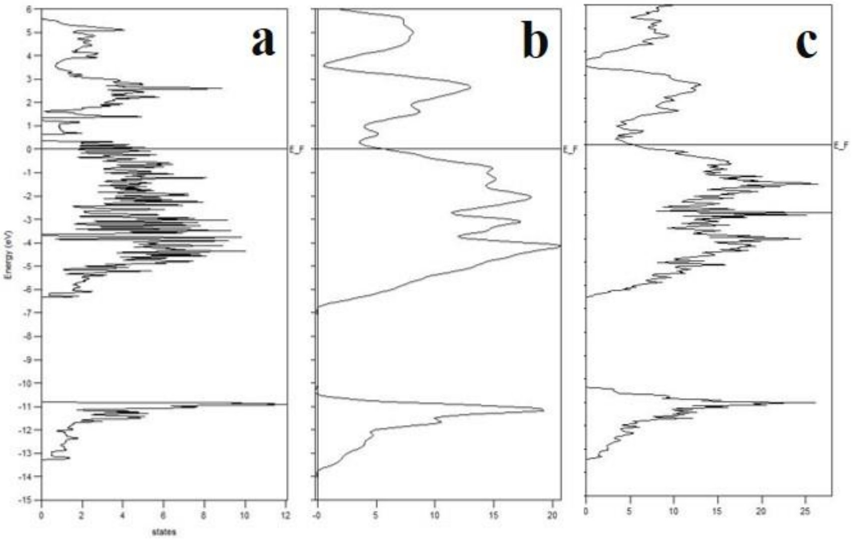

Figure S6 Comparison of total DOS for the q5 phase. a) isolated layer, M06L+SOC level of theory b) bulk, M06L+SOC level of theory c) bulk, PBE-noSOC level of theory.

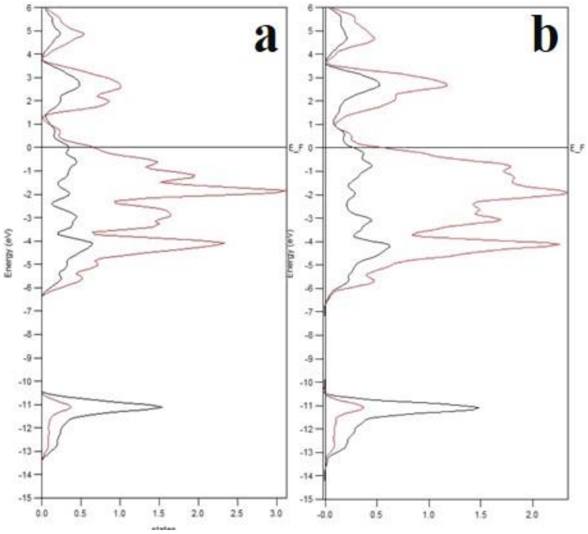

Figure S7 p-DOS of HT (a) and q5 (b) phases. The red (blue) line represents the projection onto Ir (Te) atom. For HT (a), the only symmetry-unique Ir and Te atoms are shown, whereas for q5 (b), we plot the Ir and Te atoms are those farthest from the 'dimers'. Note how the p-DOS's of HT and q5 are almost superimposable, demonstrating that the atoms far from dimers display an electronic structure very similar to the HT phase.

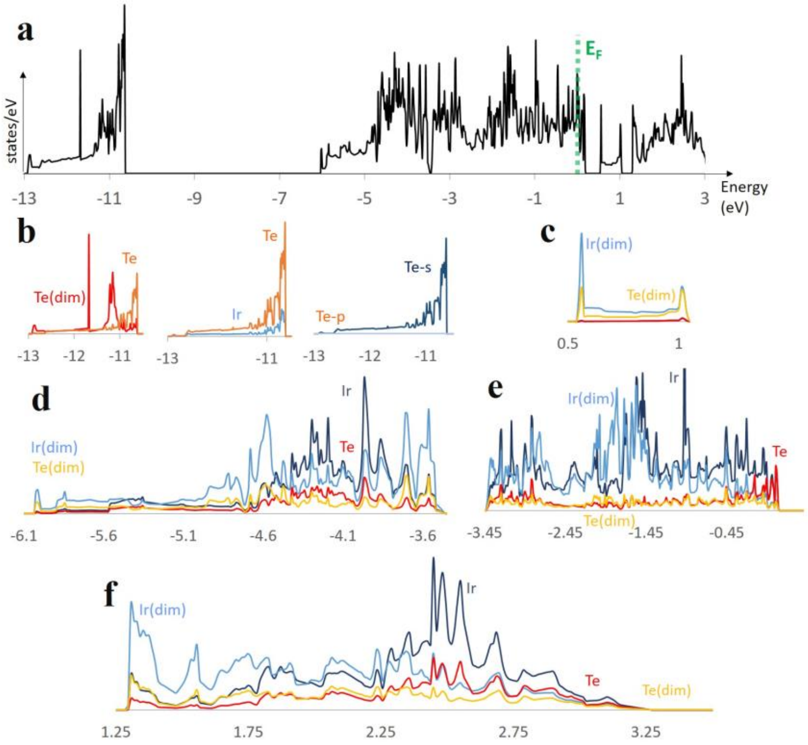

Figure S8 Density of states for an isolated layer of $IrTe_2$ in q5 phase, calculated with PBE functional (SOC neglected). Total DOS shown in black (a). Projections are shown for various DOS sections: -13/-10 (b), +0.4/+1.2 (c), -6.2/-3.5 (d), -3.5/+0.3 (e), +1.2/+3.4 (f). Orbital projections are reported only in panel *b*, while the other panels report p-DOS on atoms. Ir/Te refer to the atoms far from the dimer, while the atoms forming the dimeer are labelled as "(dim)". Note that the differences between this plot and Fig. 3 (same system at M06L+SOC level of theory) lie in the exact position and height of the peaks, rather than in the overall shape of the DOS and p-DOS. This demonstrates that the differences in the electronic structure between M06L+SOC and PBE without SOC are quantitative and not qualitative.

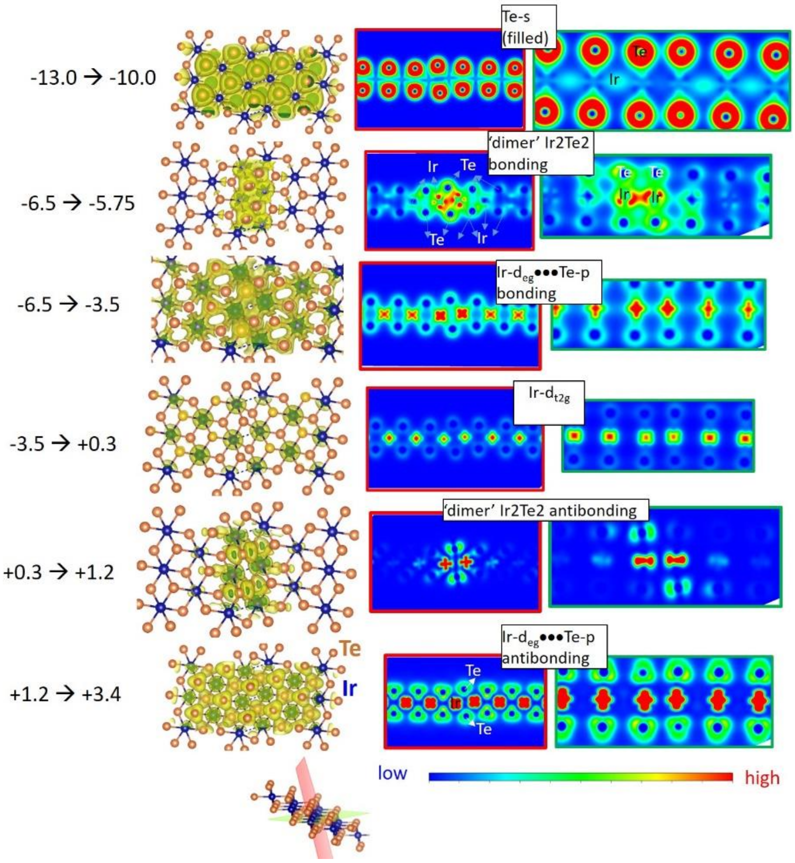

Figure S9 Charge density distribution for selected states of a single layer of the q5 phase of $IrTe_2$ calculated with PBE functional (SOC neglected). For each row, we show the charge density given by the states relative to one section of the DOS of Fig. S8. The DOS energy range adopted for each partial charge density distribution is indicated on the left. In the left column, isosurfaces of two colors are represented: green (higher value) and yellow (lower value). Ir-Ir short contacts represented as dotted blue lines. The $IrTe_2$ single layer is shows from above. On the right, the corresponding charge densities are mapped onto 2 planes, whose position is shown at the bottom of the picture. The map framed in red corresponds to the red plane shown at the bottom, and the same goes for green. Note that the internuclear axis corresponding to the shortest Ir-Ir bond ('dimer') always lies in the plane.

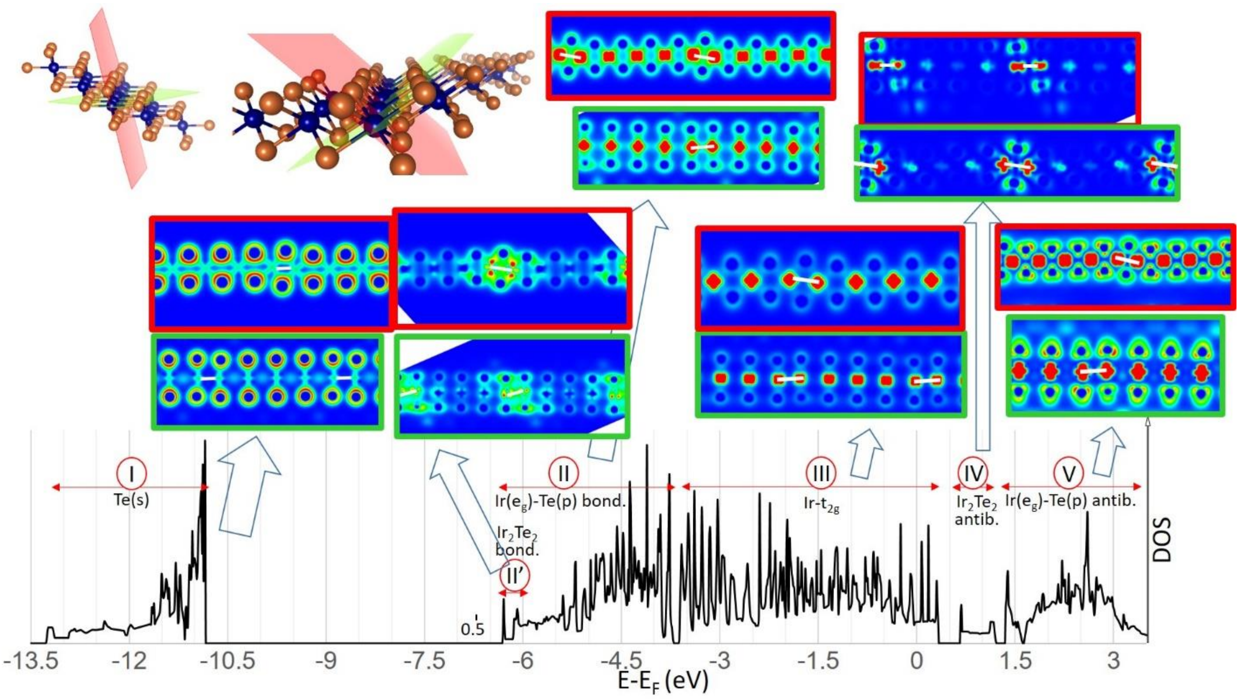

Figure S10 2-dimensional maps of partial charge density distributions for an isolated layer of $IrTe_2$ at the M06L+SOC level of theory. The DOS is the same as Fig. 3 of the main text, except that in Fig. 3 a Gaussian broadening has been added (convolution with a normalized Gaussian). Each map is the charge density calculated including only the states in a given energy range of the DOS. The energy ranges are those defined in Fig. 3 of the main text, whose DOS is reported here for convenience. For each of those ranges, 2 maps are shown: a red plane and a green plane, whose positions in the $IrTe_2$ layer can be seen in the top left images. As both planes contain the interatomic axis of the short Ir-Ir contacts, the corresponding contact is indicated in the map by a white line. The color scale ranges from blue (0), to red (highest value) through green (middle value), as in Fig. S9.

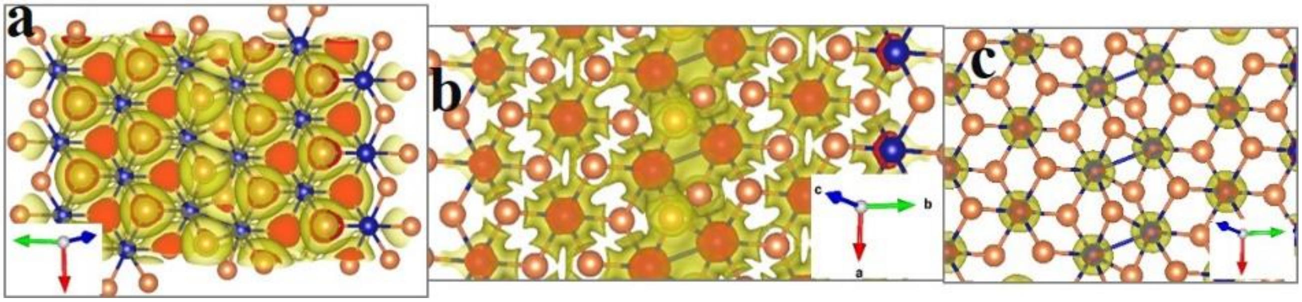

Figure S11 Charge density distribution of selected states of $IrTe_2$ in q5 phase (isolated layer), calculated at the M06L+SOC level of theory. The plotted isosurfaces represent the distribution of charge density relative to DOS sections in Fig. S10 (and Fig. 3 of the main text): range from -12 to -10 eV (i.e. most of region I) (a), region II (b), and region III (c). Note that (b) and (c) are simply wider views of Figs 4c and 4d of the main text. We report in the following the isovalues adopted here and in Fig. 4 of the main text (values in parenthesis are for the red isosurface). a) 0.002 (0.01) ; b and fig. 4c) 0.009 (0.03) ; c and Fig. 4d) 0.04 (0.16) ; Fig. 4a) 0.0015 (0.0025); Fig. 4b) 0.002 (0.005). The units of for the isosurfaces are those adopted by VASP, i.e. electrons/Å^3 multiplied by the volume of the unit cell (798.73 Å^3).

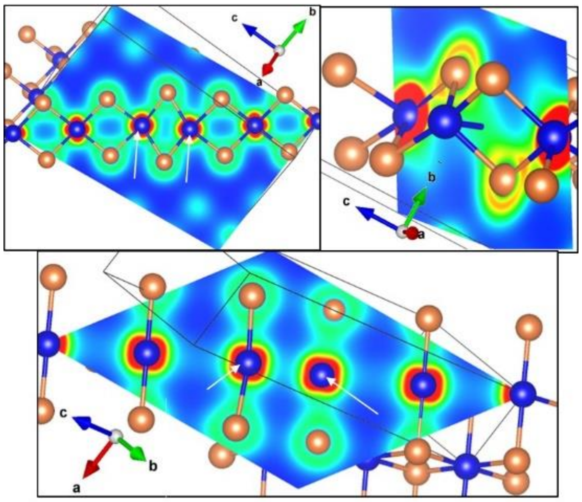

Figure S12 Total charge density distribution of bulk $IrTe_2$, to be interpreted in the QTAIM framework. Charge density is color-mapped onto 3 planes passing through the middle of the short Ir-Ir 'bond'. This bond ('dimer') is indicated by a blue line in the top right panel, while in the other two panels the Ir atoms forming the dimer are indicated by a white arrow. The color scale (blue-green-red) is 0.0-0.01 for the top left panel and 0.0-0.02 for the other two, in vasp unit (Volume of the unit cell 399.40 Å^3; see Fig. S11).

The Quantum Theory of Atoms in Molecules (QTAIM[18]) shows that whenever a chemical bond is present, a 'bond path' appears in the electron density distribution. The bond path is a line joining two nuclei, along which the charge density is a maximum with respect to all directions perpendicular to the path. While bond paths are found between all nearest neighbors Ir-Te contacts (not shown), these plots show that there are no Ir-Ir bond paths. In fact, the mid-point between the two Ir is a saddle point (maximum in two directions, minimum in the third one, that is the direction joining the two Ir nuclei), or a 'ring critical point' in QTAIM language. Therefore, within the QTAIM framework, there is no Ir-Ir direct bond. These evidences support the fact that the 'dimer' is actually a multicenter bond involving Te atoms (see discussion in the main text).

S4 Results of Se-doping

Table S2. Energetics of Se doping.

HT→q5 $IrTe_{1.90}Se_{0.10}$			HT→q6 $IrTe_{1.96}Se_{0.04}$	
Se position[b]	ΔE[a] [meV/atom]		Se position[b]	ΔE[a] [meV/atom]
dimer	-15.5 (-10.2[c])		dimer	-29.8 (-9.8[c])
next to dimer	-4.0 (-3.6[c])		next to dimer	-28.3 (-7.6[c])
far from dimer	-4.7 (-4.5[c])			

[a] ΔE=E(qn)-E(HT). For comparison the values for the undoped compound are -13.51 (-6.04) and -24.04 (-8.88) meV/atom for the q5 and q6 phases, respectively, adopting the M06L+SOC (PBE-noSOC).

[b] See Section S1.3 for a description of the various substitution positions. "dimer" indicates that the Te atom that has been substituted is the one bridging the two Ir atoms forming the 'dimers', *e.g.* Te2 in Fig. 5a of the main text.

[c] values in parenthesis were obtained with PBE neglecting SOC

Table S3. Representative COHP values in pure and Se-doped IrTe2 in q5 phase

bond	ICOHP [eV]	ΔICOHP [eV][a]	Δd [Å][a]
Ir-Ir, shortest ($IrTe_{1.90}Se_{0.10}$)	-0.56	-0.42	-0.96
Ir-Ir, shortest ($IrTe_2$)	-0.50	-0.37	-0.84
Ir-Se, shortest ($IrTe_{1.90}Se_{0.10}$)	-2.96	-0.34	-0.07
Ir-Te, shortest ($IrTe_2$)	-2.81	-0.26	-0.06

[a] change with respect to HT phase (*e.g.* d(q5)-d(HT))

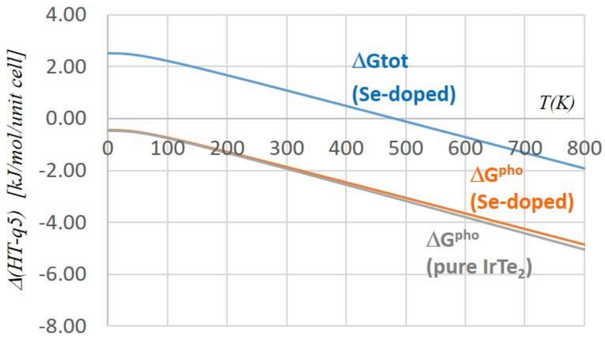

Figure S13 Theormodynamics of the HT → q5 transition of $IrTe_{1.90}Se_{0.10}$, obtained at the PBE level of theory (SOC neglected). Note how the transition temperature is much higher with respect to the undoped compound (Fig. S3), in agreement with experimental observations (see main text). Beside showing the total free energy of the reaction (blue), we compare the vibrational free energy of the Se-doped compound (orange) to that of undoped IrTe2 (grey). This comparison shows that the q5 phase is stabilized in the doped compound not by the vibrational contributions, which is almost identical to that of the undoped compound, but rather by the electronic energy term (Table S3).

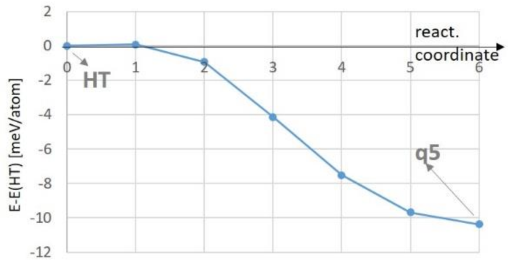

Figure S14 Nudged Elastic Bands results for the HT → q5 transition of $IrTe_{1.90}Se_{0.10}$, obtained at the PBE level of theory (SOC neglected). The energetics of the transition path from HT to q5 phases is obtained through a 7 images CI-NEB calculation (see Section S1 for details).

REFERENCES

[1] G. Kresse and J. Furthmüller "Efficiency of ab-initio total energy calculations for metals and semiconductors using a plane-wave basis set" Computational Materials Science **6**, 15 (1996).
[2] P. E. Blöchl "Projector augmented-wave method." Phys. Rev. B **50**, 17953–17979 (1994).
[3] https://cms.mpi.univie.ac.at/vasp/vasp/Recommended_PAW_potentials_DFT_calculations_using_vasp_5_2.html

[4] G. L. Pascut, T. Birol, M. J. Gutmann, J. J. Yang, S.-W.Cheong, K. Haule, and V. Kiryukhin "Series of alternating states with unpolarized and spin-polarized bands in dimerized $IrTe_2$" Phys. Rev. B **90**, 195122 (2014).
[5] J. P. Perdew, K. Burke, and M. Ernzerhof "Generalized gradient approximation made simple" Phys. Rev. Lett. **77**, 3865 (1996).
[6] G. I. Csonka, J. P. Perdew, A. Ruzsinszky, P. H. T.Philipsen, S. Lebegue, J. Paier, O. A. Vydrov, and J. G. Angyan "Assessing the performance of recent density functionals for bulk solids." Phys. Rev. B **79**, 155107 (2009).
[7] J. P. Perdew and K. Schmidt. "Jacob's ladder of density functional approximations for the exchange-correlation energy." *AIP Conference Proceedings*. Vol. 577. No. 1. American Institute of Physics (2001).
[8] Y. Zhao and D. G. Truhlar "A new local density functional for main-group thermochemistry, transition metal bonding, thermochemical kinetics, and noncovalent interactions." The Journal of Chemical Physics **125**, 194101 (2006)
[9] Y. S. Oh, J. J. Yang, Y. Horibe, and S.-W. Cheong "Anionic depolymerization transition in $IrTe_2$" Phys. Rev. Lett. **110**, 127209 (2013).
[10] A. Togo and I. Tanaka "First principles phonon calculations in materials science" Scripta Materialia **108**, 1 (2015).
[11] http://theory.cm.utexas.edu/vtsttools/scripts.html
[12] G. Henkelman and H. Jónsson "Improved tangent estimate in the nudged elastic band method for finding minimum energy paths and saddle points." J. Chem. Phys. **113**, 9901-9904 (2000)
[13] H. Eyring "The activated complex in chemical reactions." J. Chem. Phys. **3**, 107 (1935)
[14] G. H. Vineyard "Frequency factors and isotope effects in solid state rate processes." J. Phys. Chem. Solid, **3**, 121-127 (1953).
[15] S. Maintz, V. L. Deringer, A. L. Tchougreeff, R. Dronskowski "LOBSTER: A tool to extract chemical bonding from plane-wave based DFT." *J. Comput. Chem.* **2016**, *37*, 1030—1035.
[16] R. Dronskowski and P. E. Bloechl "Crystal orbital Hamilton populations (COHP): energy-resolved visualization of chemical bonding in solids based on density-functional calculations." The Journal of Physical Chemistry **97**, 8617 (1993).
[17] V. L. Deringer, A. L. Tchougreeff, and R. Dronskowski "Crystal orbital Hamilton population (COHP) analysis as projected from plane-wave basis sets." The Journal of Physical Chemistry A **115**, 5461 (2011).
[18] R. F. W. Bader *Atoms in Molecules: A Quantum Theory* (Oxford University Press, 1990).
